# Supplementary material for: Cryo-EM structures of prokaryotic ligand-gated ion channel GLIC provide insights into gating in a lipid environment
Source: Nat Commun. 2024 Apr 5;15:2967. doi: 10.1038/s41467-024-47370-w (PMC10997623; doi:10.1038/s41467-024-47370-w)
Supplement: Supplementary file 1 — Supplementary Information [file 41467_2024_47370_MOESM1_ESM.pdf]

Supplementary Information

Cryo-EM structures of prokaryotic ligand-gated ion channel GLIC provide insights into gating in a lipid environment

Nikhil Bharambe[1*], Zhuowen Li[1*], David Seiferth[2], Asha Manikkoth Balakrishna[1], Philip C. Biggin[2], and Sandip Basak[1, 3#]

[1]School of Biological Sciences, Nanyang Technological University, Singapore 637551

[2]Structural Bioinformatics and Computational Biochemistry, Department of Biochemistry, University of Oxford, Oxford, UK

[3]NTU Institute of Structural Biology, Nanyang Technological University, Singapore 639798

∗These authors contributed equally to this work.

[#]Corresponding author: Sandip Basak, Email: sandip.basak@ntu.edu.sg

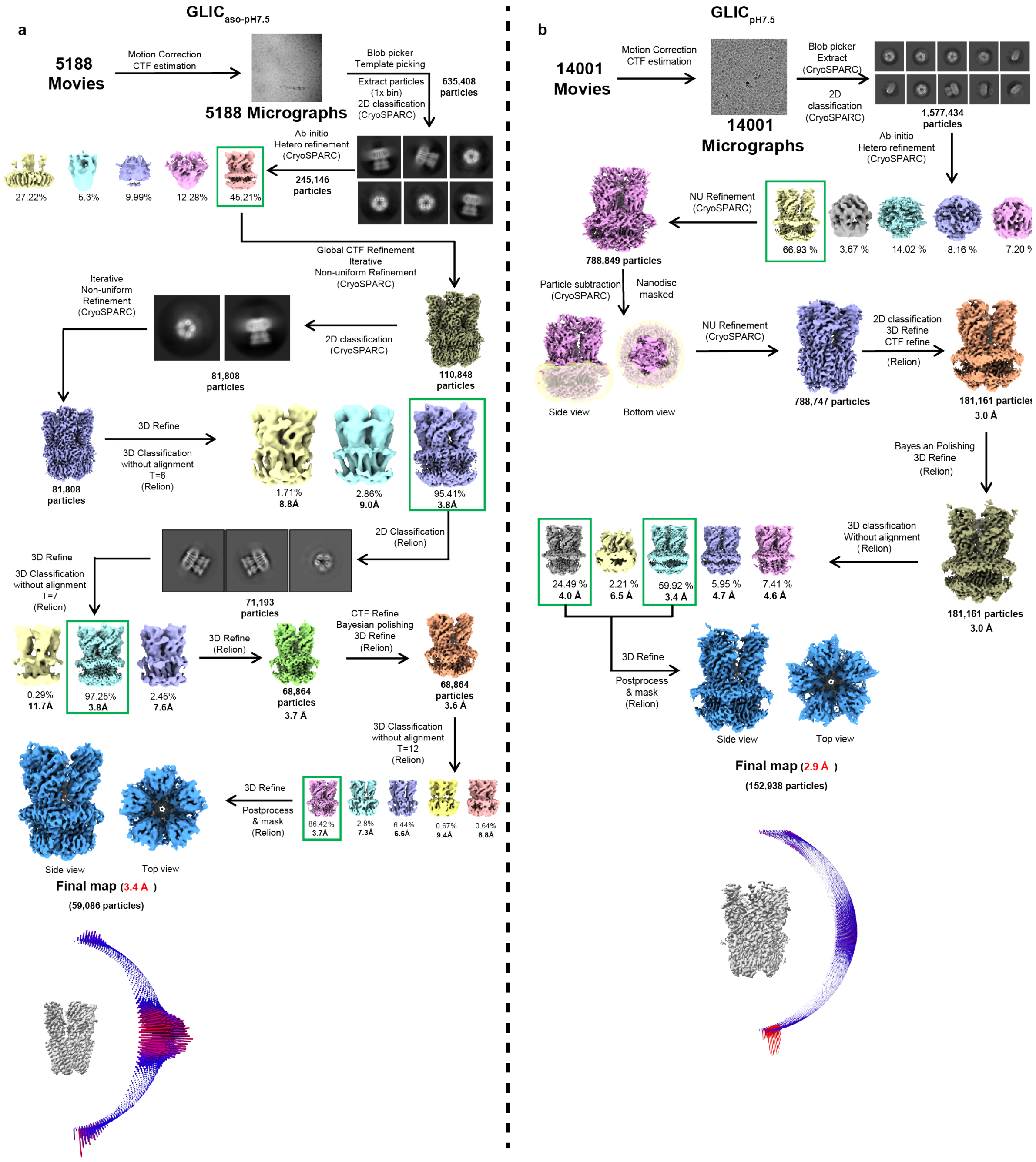

Supplementary figure 1. Cryo-EM data processing workflow of GLIC in the apo state. Cryo-EM data processing workflow of **a** GLIC$_{aso\text{-}pH7.5}$ and **b** GLIC$_{pH7.5}$ showing representative micrograph and 2D classes and angular distribution. Systematic data processing, including iterative 2D, 3D classification and refinement, led to nominal resolutions of 3.4 Å and 2.9 Å map for GLIC$_{aso\text{-}pH7.5}$ and GLIC$_{pH7.5}$, respectively.

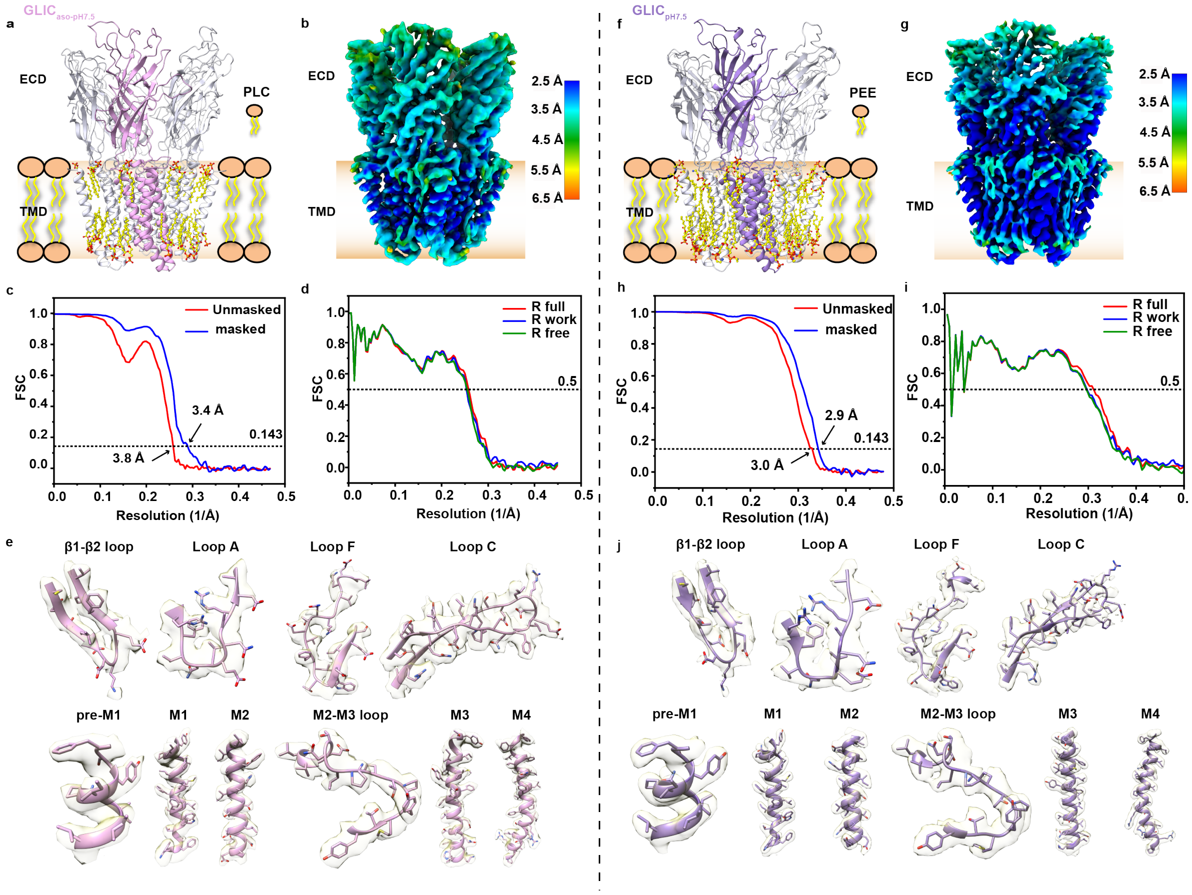

Supplementary figure 2. Cryo-EM data quality assessment and model validation of GLIC reconstituted in nanodiscs at pH 7.5. a, f Overall structure of GLIC$_{aso\text{-}pH7.5}$ (plum) and GLIC$_{pH7.5}$ (purple). One subunit is colored, whereas the other subunits are in gray. Lipids (PLC: phosphatidylcholine; PEE: phosphatidylethanolamine) are colored in yellow and shown as balls and sticks. Membrane bilayers are shown as a schematic representation. **b, g** The side view of 3D reconstructed maps of corresponding structures are colored by the local resolution calculated using the ResMap program. A color key representing resolution is also inserted. A schematic of the bilayer is shown as a gradient of apricot color. **c, h** The gold standard Fourier shell correlation (FSC) curves before (red) and after (blue) using the mask are shown for the respective structures. The dashed line represents a FSC at 0.143. **d, i** Model Vs map validation FSC curves of the corresponding structure are shown. For cross validation of model calculation, the FSC curves of the refined model versus the summed map (R_{full}, red), refined model versus half map 1 (used during refinement, R_{work}, blue), and refined model versus half map 2 (not used during refinement, R_{free}, green) are plotted. **e, j** Validation of various regions is shown by extracting density maps of critical regions (transparent volume in yellow). The corresponding region of the model is depicted as a cartoon with sticks.

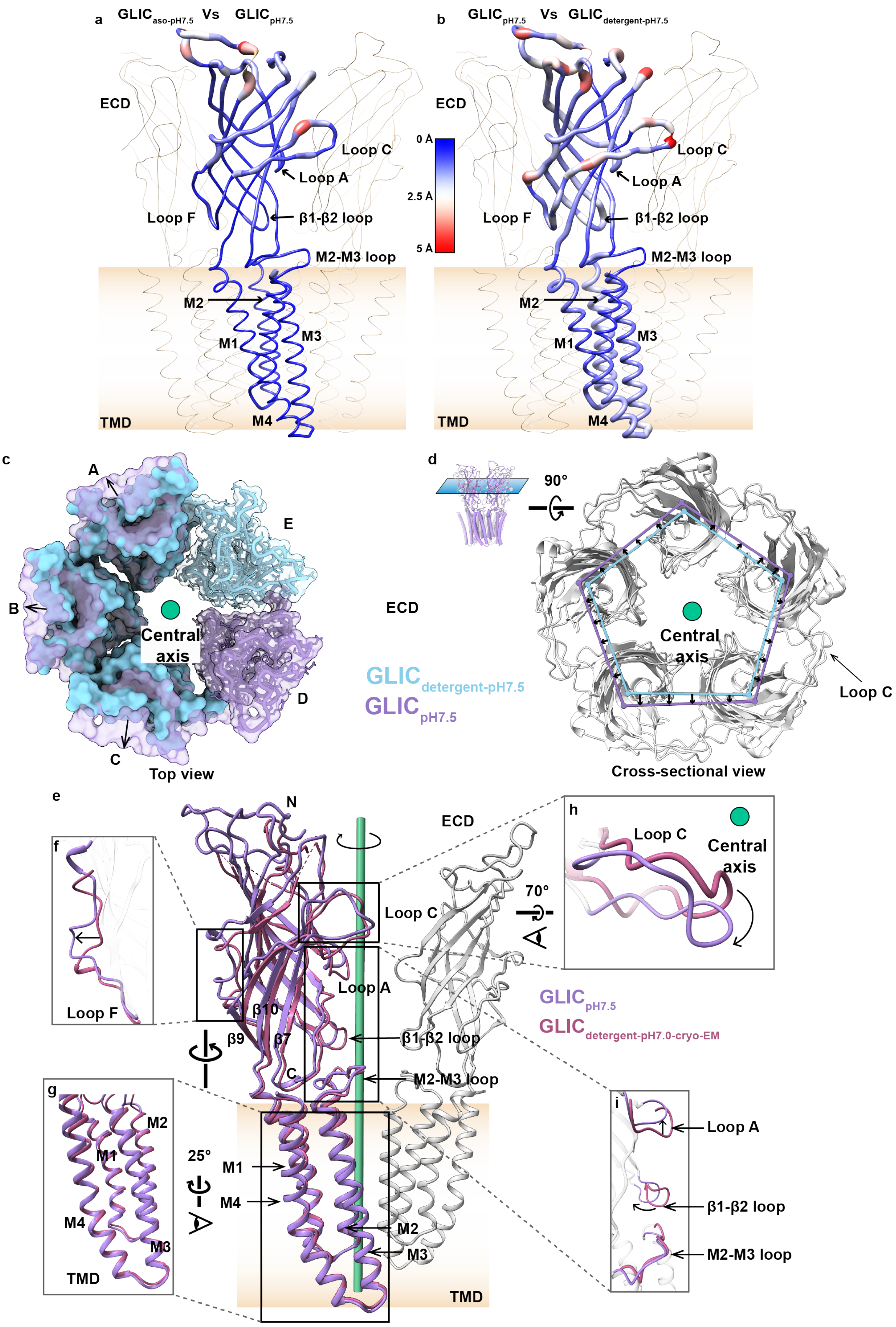

Supplementary figure 3. Comparison of GLIC in various apo states. The RMSD of backbone (Cα) is calculated. Pairwise comparisons of conformational changes are shown for **a** $GLIC_{aso\text{-}pH7.5}$ Vs $GLIC_{pH7.5}$ and **b** $GLIC_{pH7.5}$ Vs $GLIC_{detergent\text{-}pH7.5}$. The principal subunit is

represented as putty and colored by RMSD. Other subunits are represented in licorice (tan). The RMSD color code and ribbon thickness scale is inserted. **c** Top view (from extracellular side) of ECD shows expansion in $GLIC_{pH7.5}$ (purple) compared to $GLIC_{detergent\text{-}pH7.5}$ (skyblue). The subunits A–C of both structures are superimposed and shown in the surface representation. For clarity, the $GLIC_{pH7.5}$ surface is made transparent. Subunits D and E are shown only for $GLIC_{pH7.5}$ and $GLIC_{detergent\text{-}pH7.5}$, respectively, in the transparent surface, licorice, and stick representations. Arrows indicate the expansion of ECD in $GLIC_{pH7.5}$ (purple) compared with the crystal structure. The central axis is shown in green. **d** Cross-sectional view showing the overall expansion of ECD in $GLIC_{pH7.5}$ compared with $GLIC_{detergent\text{-}pH7.5}$ indicated by arrows. The centroid of ECD was calculated for each subunit and neighboring centroid are connected to demonstrate the average expansion in the ECD. The centroids and axes are colored according to the individual structure. **e** Superposition of $GLIC_{pH7.5}$ (purple) and $GLIC_{detergent\text{-}pH7.0}$-cryo-EM (china rose) shows conformational differences in Loop F (**f**), TMD (**g**), Loop C (**h**) Loop A, β1-β2 loop, and M2–M3 loop (**i**), which are highlighted in zoom-in views. Only one subunit is colored respective to the structure and the diagonal subunit is colored in gray. The membrane bilayer is shown as a gradient of apricot color. The central axis is shown as a green cylinder or circle. Arrows indicate the direction of movement. The viewing angle is given (wherever applicable) along with the symbol.

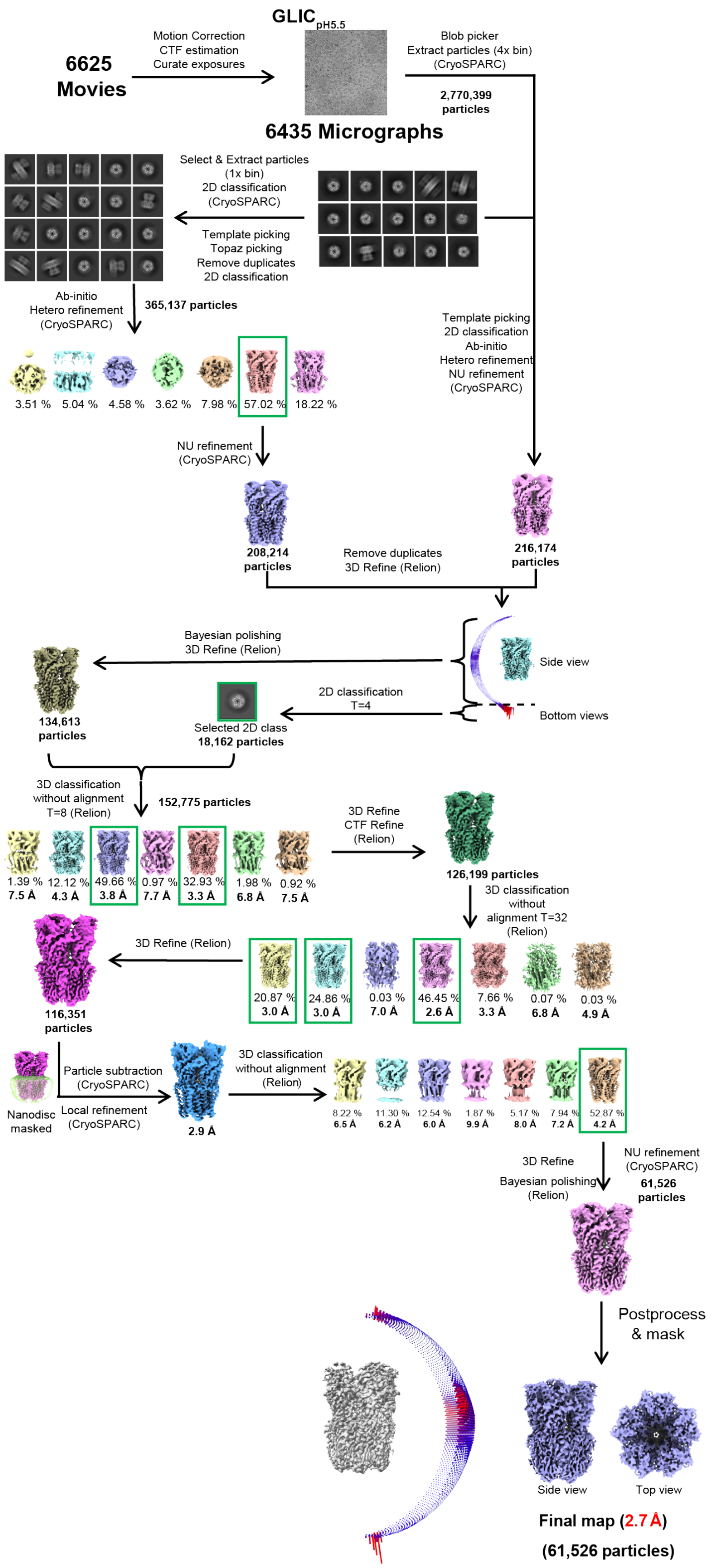

Supplementary figure 4. Cryo-EM data processing workflow of GLIC at pH 5.5. Cryo-EM data processing workflow of $GLIC_{pH5.5}$ showing representative micrograph and 2D

classes and angular distribution. Systematic data processing, including iterative 2D, 3D classification and refinement, led to a nominal resolution of 2.7 Å.

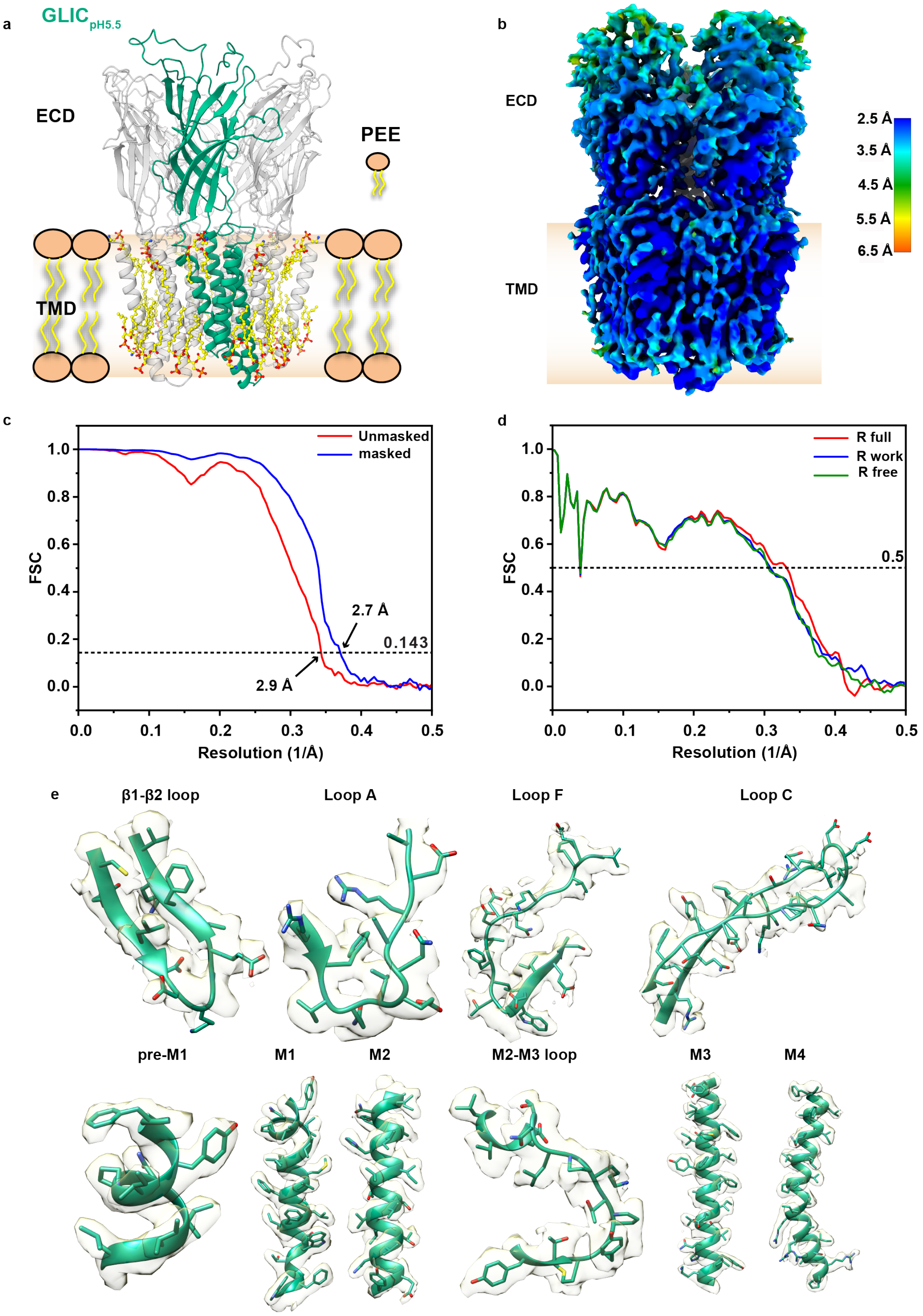

Supplementary figure 5. Cryo-EM data quality assessment and model validation of GLIC reconstituted in nanodiscs at pH 5.5. a The overall structure of $GLIC_{pH5.5}$. One subunit is colored green and the other subunits are colored gray. Lipids (PEE:

phosphatidylethanolamine, yellow) are shown as balls and sticks and as schematics in the membrane bilayer. **b** The side view of 3D reconstructed map of the corresponding structure is colored by the local resolution calculated using the ResMap program. A color key corresponding to the resolution is also inserted. A schematic of the bilayer is shown as a gradient of apricot color. **c** The gold standard Fourier shell correlation (FSC) curves before (red) and after (blue) using the mask are shown for the respective structure. The dashed line represents FSC of 0.143. **d** Model Vs map validation FSC curves for the corresponding structure are shown. For cross validation of model calculation, the FSC curves of the refined model versus summed map (R_{full}, red), refined model versus half map 1 (used during refinement, R_{work}, blue), and refined model versus half map 2 (not used during refinement, R_{free}, green) are plotted. **e** Validation of various regions is shown by extracting density maps of critical regions (transparent volume in yellow). The corresponding region of the model is depicted as a cartoon with sticks.

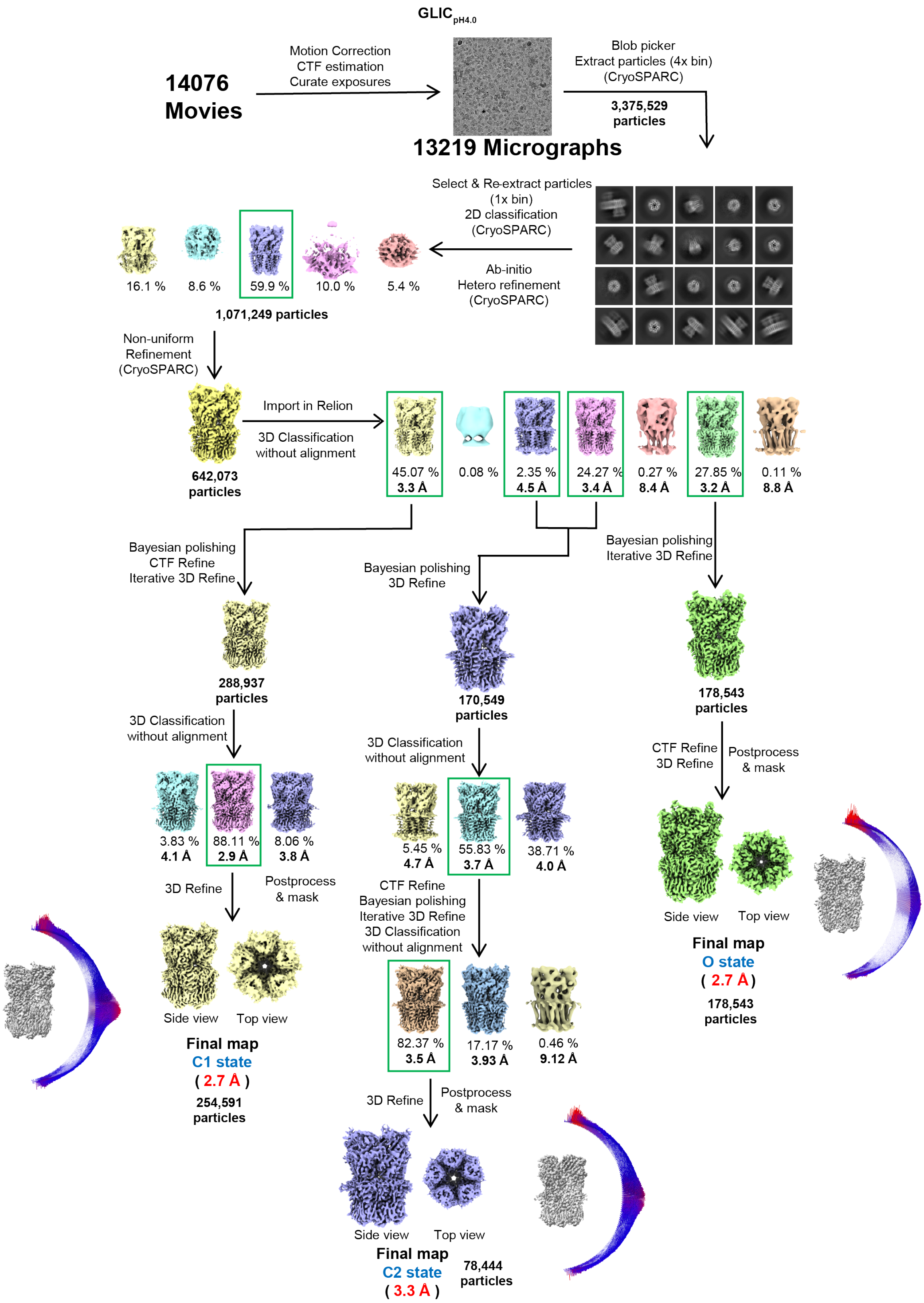

Supplementary figure 6. Cryo-EM data processing workflow of GLIC at pH 4.0. Cryo-EM data processing workflow of $GLIC_{pH4.0}$ showing representative micrograph and 2D classes and angular distribution. Systematic data processing, including iterative 2D, 3D

classification and refinement, led to three states C1, C2 and O with nominal resolutions of 2.7 Å, 3.3 Å, and 2.7 Å, respectively. The population distribution of C1, C2, and O states was found to be 50%, 15%, and 35%, respectively.

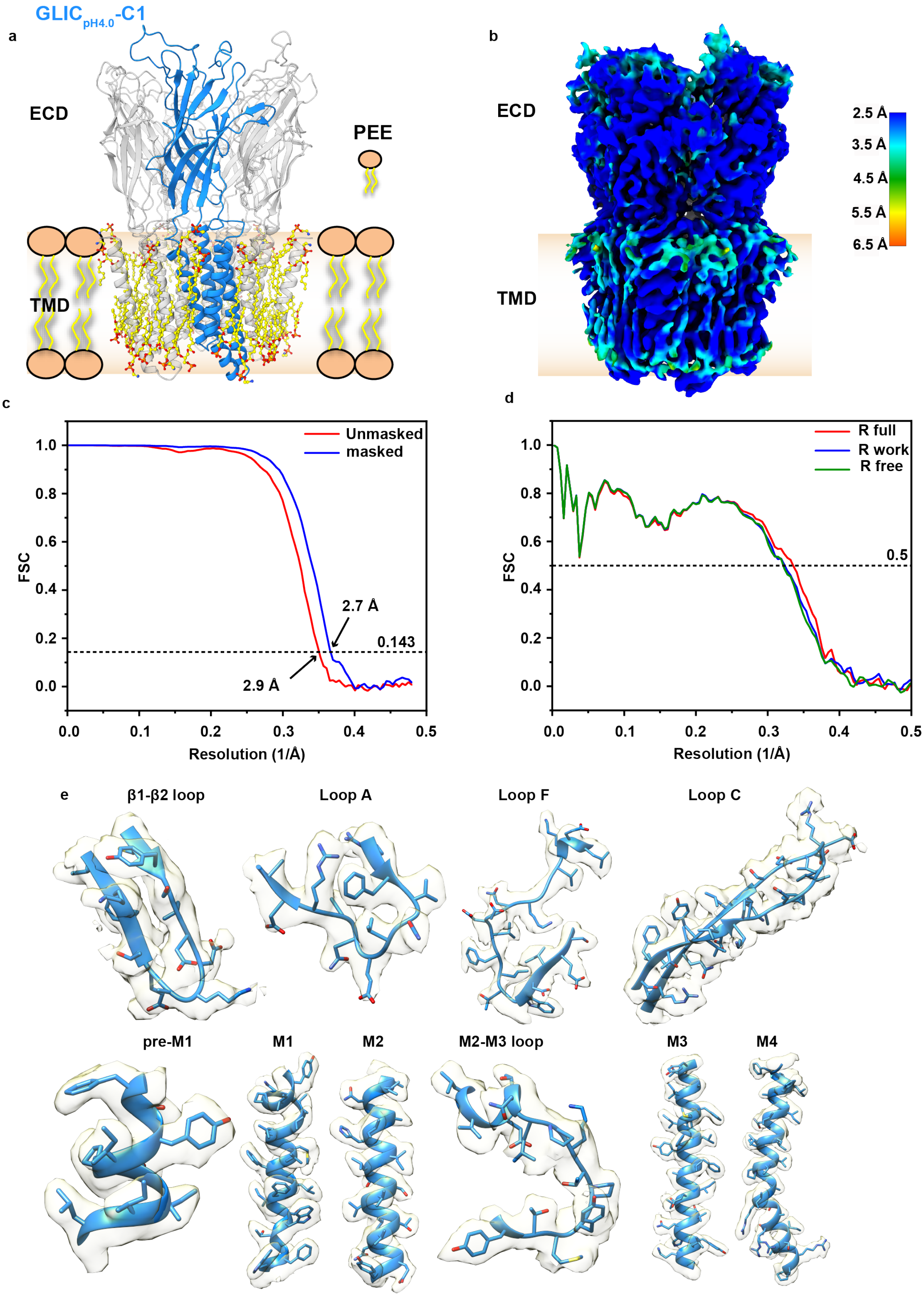

Supplementary figure 7. Cryo-EM data quality assessment and model validation of GLIC reconstituted in nanodiscs at pH 4.0 in state C1. A Overall structure of GLIC$_{pH4.0}$-C1. One subunit is colored dodger blue, whereas the other subunits are colored gray. Lipids

(PEE: phosphatidylethanolamine, yellow) are shown as balls and sticks and as schematics in the membrane bilayer. **b** Side view of 3D reconstructed map of the corresponding structure is colored by the local resolution calculated using the ResMap program. A color key corresponding to the resolution is also inserted. A schematic of the bilayer is shown as a gradient of apricot color. **c** The gold standard Fourier shell correlation (FSC) curves before (red) and after (blue) using the mask are shown for the respective structures. The dashed line represents an FSC of 0.143. **d** Model Vs map validation FSC curves for the corresponding structure are shown. For cross validation of model calculation, the FSC curves of the refined model versus the summed map (R_{full}, red), refined model versus half map 1 (used during refinement, R_{work}, blue), and refined model versus half map 2 (not used during refinement, R_{free}, green) are plotted. **e** Validation of various regions is shown by extracting density maps of critical regions (transparent volume in yellow). The corresponding region of the model is depicted as a cartoon with sticks.

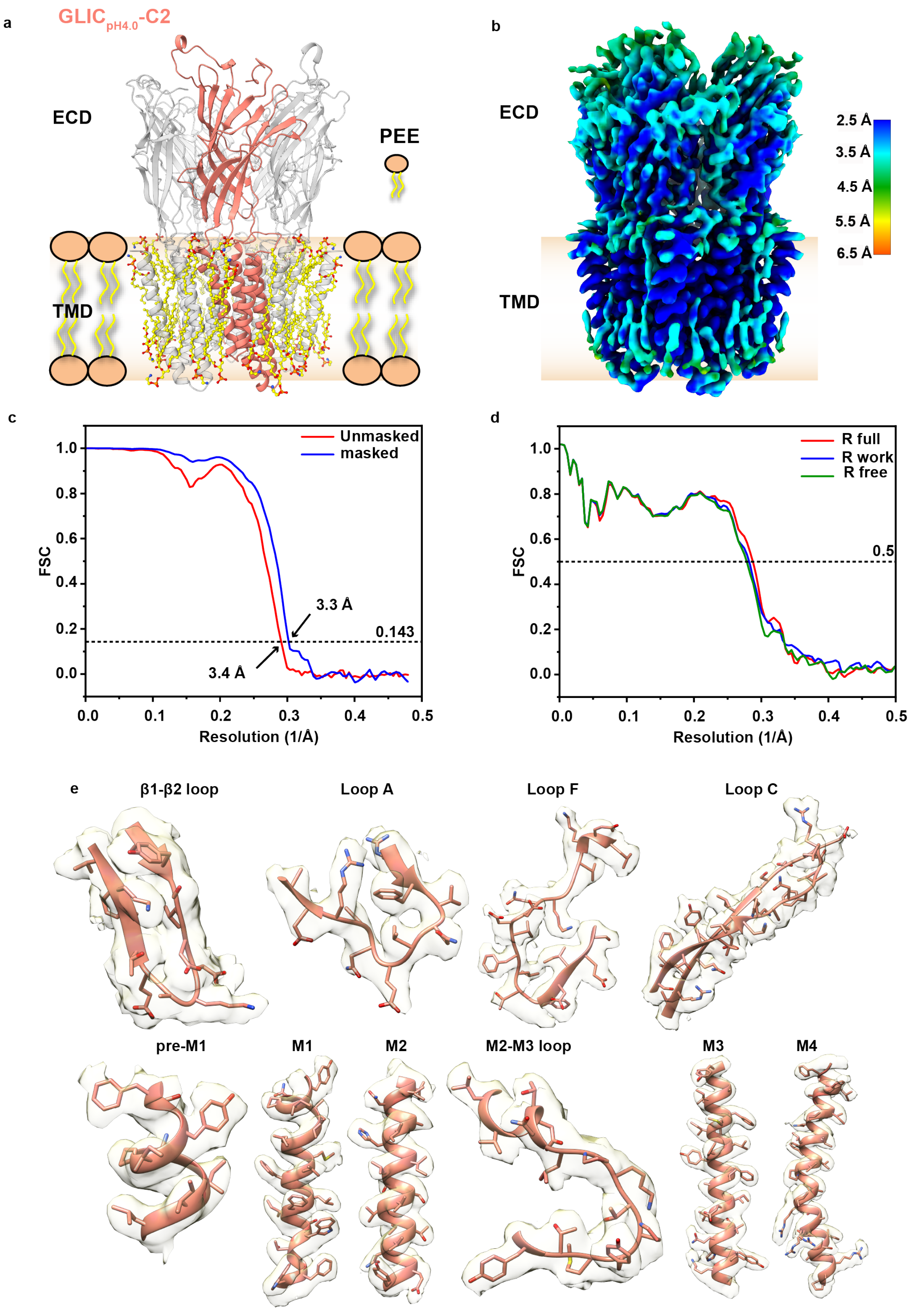

Supplementary figure 8. Cryo-EM data quality assessment and model validation of GLIC reconstituted in nanodiscs at pH 4.0 in state C2. a Overall structure of $GLIC_{pH4.0}$-C2. One subunit is colored salmon and the other subunits are colored gray. Lipids (PEE:

phosphatidylethanolamine, yellow) are shown as balls and sticks and as schematics in the membrane bilayer. **b** The side view of 3D reconstructed map of corresponding structure is colored by the local resolution calculated using the ResMap program. A color key corresponding to the resolution is also inserted. A schematic of the bilayer is shown as a gradient of apricot color. **c** The gold standard Fourier shell correlation (FSC) curves before (red) and after (blue) using the mask are shown for the respective structures. The dashed line represents an FSC of 0.143. **d** Model Vs map validation FSC curves for the corresponding structure are shown. For cross validation of model calculation, the FSC curves of the refined model versus the summed map (R_{full}, red), refined model versus half map 1 (used during refinement, R_{work}, blue), and refined model versus half map 2 (not used during refinement, R_{free}, green) are plotted. **e** Validation of various regions is shown by extracting density maps of critical regions (transparent volume in yellow). The corresponding region of the model is depicted as a cartoon with sticks.

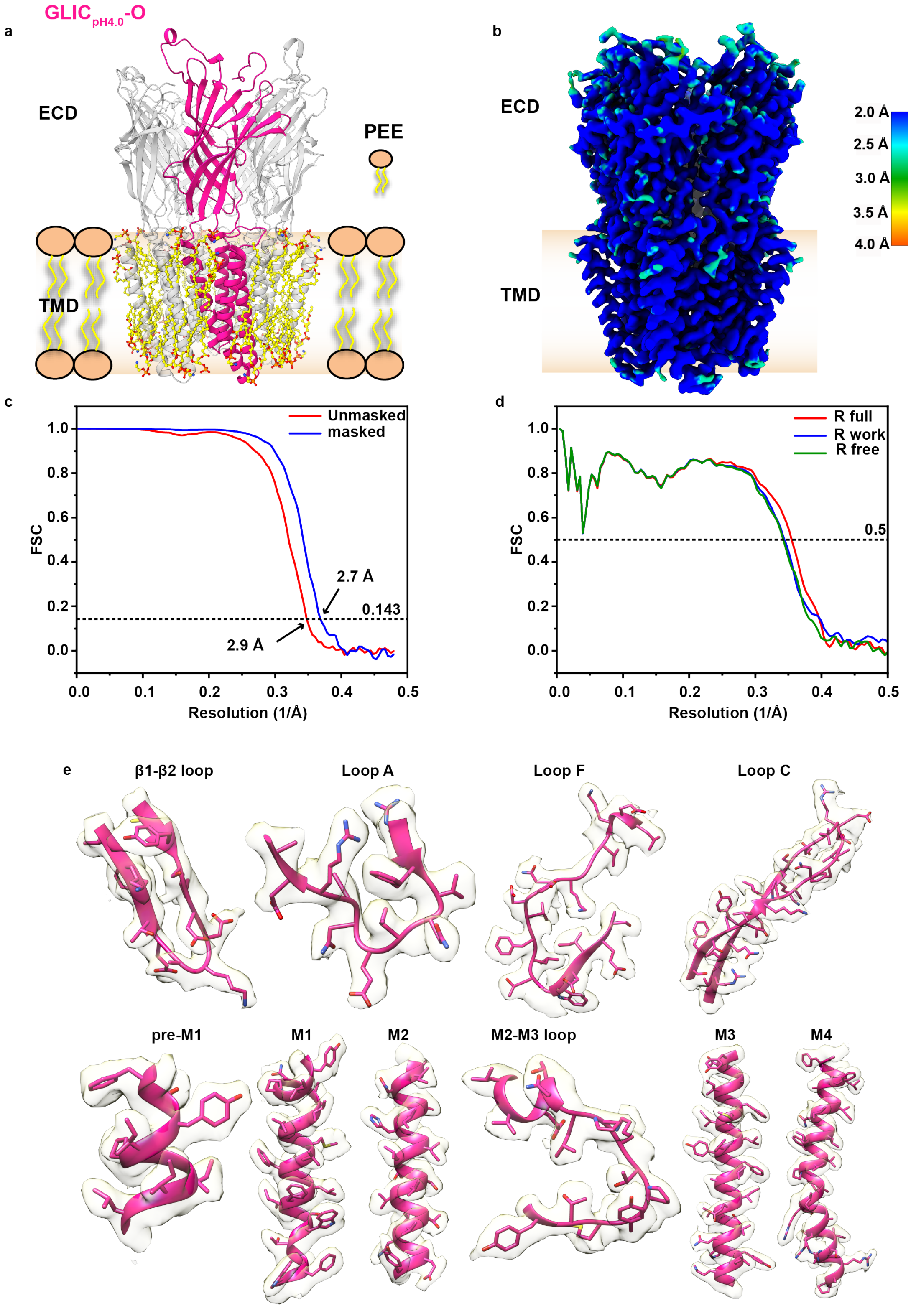

Supplementary figure 9. Cryo-EM data quality assessment and model validation of GLIC reconstituted in nanodiscs at pH 4.0 in state O. a Overall structure of $GLIC_{pH4.0}$-O. One subunit is colored deep pink and the other subunits are colored gray. Lipids (PEE:

phosphatidylethanolamine, yellow) are shown as balls and sticks and as schematics in the membrane bilayer. **b** Side view of 3D reconstructed map of the corresponding structure is colored by the local resolution calculated using the ResMap program. A color key corresponding to the resolution is also inserted. A schematic of the bilayer is shown as a gradient of apricot color. **c** The gold standard Fourier shell correlation (FSC) curves before (red) and after (blue) using the mask are shown for the respective structures. The dashed line represents an FSC of 0.143. **d** Model Vs map validation FSC curves for the corresponding structure are shown. For cross validation of model calculation, the FSC curves of the refined model versus the summed map (R_{full}, red), refined model versus half map 1 (used during refinement, R_{work}, blue), and the refined model versus half map 2 (not used during refinement, R_{free}, green) are plotted. **e** Validation of various regions is shown by extracting density maps of critical regions (transparent volume in yellow). The corresponding region of the model is depicted as a cartoon with sticks.

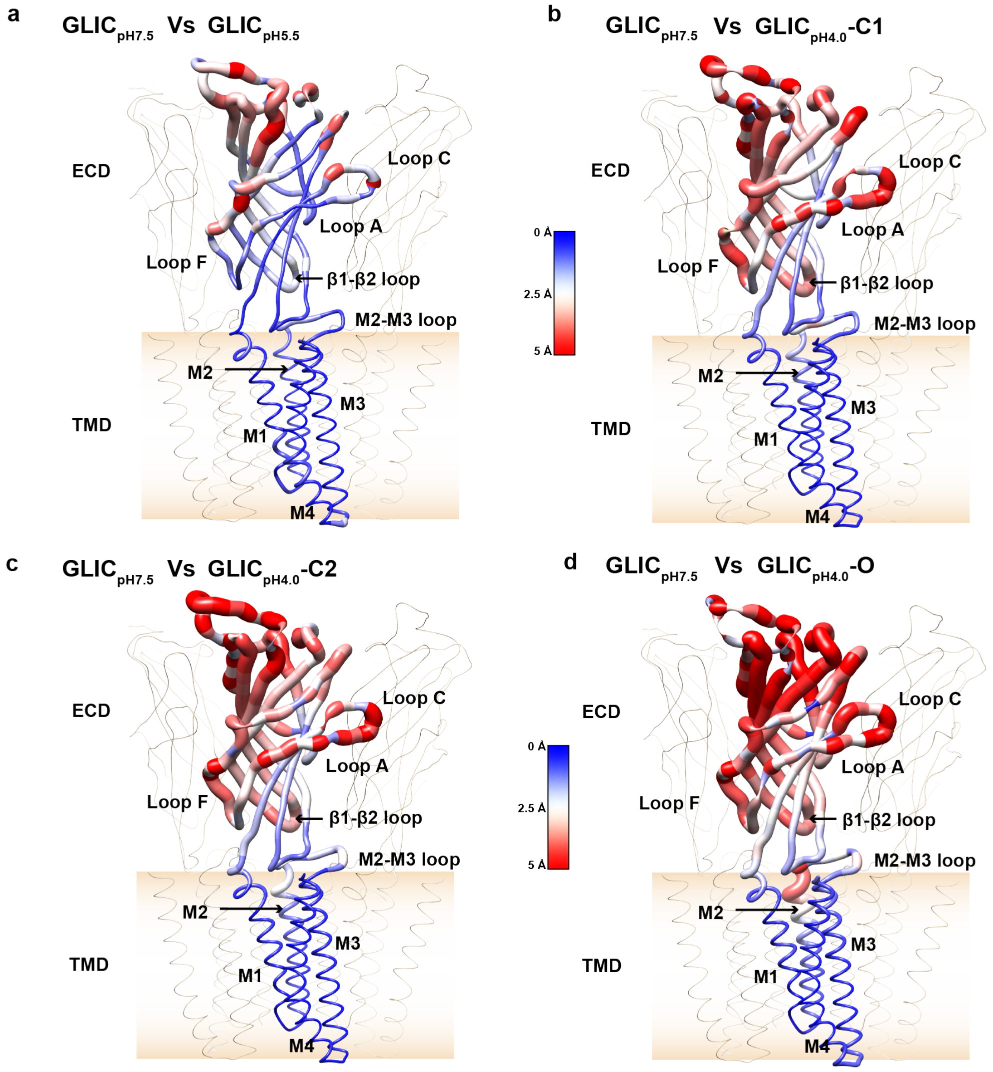

Supplementary figure 10. Pairwise comparison of GLIC structures. Pairwise comparisons of GLIC$_{pH7.5}$ with **(a)** GLIC$_{pH5.5}$, **(b)** GLIC$_{pH4.0}$-C1, **(c)** GLIC$_{pH4.0}$-C2, and **(d)** GLIC$_{pH4.0}$-O show gradual conformational changes, particularly in the ECD, upper part of M2 helix and M2–M3 loop. The principal subunit is represented as putty and is colored by RMSD. Other subunits are represented in licorice (tan). The RMSD color code and ribbon thickness scale are inserted. The membrane bilayer is shown as a gradient of apricot color.

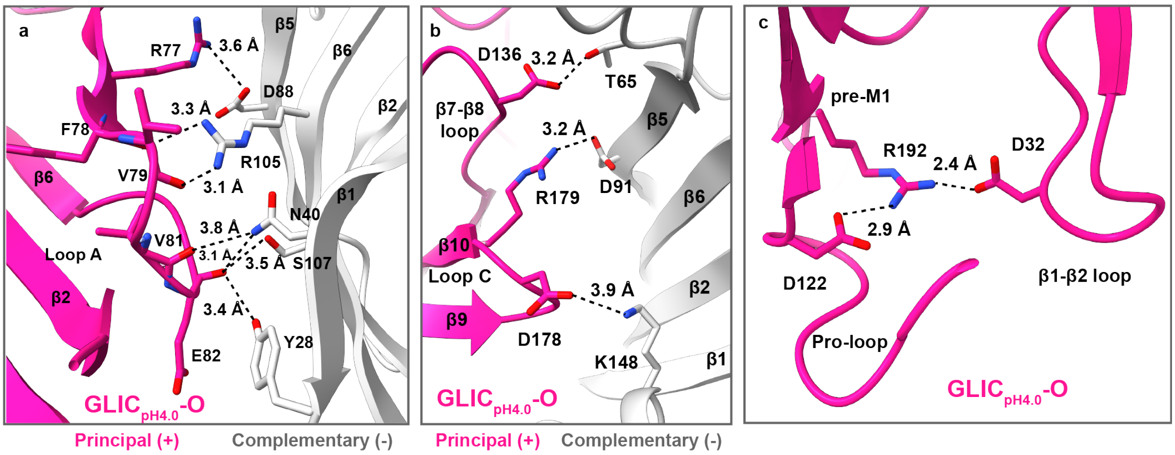

Supplementary figure 11. Intersubunit interactions in GLIC$_{pH4.0}$-O. The residues in the principal (+) subunit (deep pink) of GLIC$_{pH4.0}$-O interact with the residues present in the complementary (-) subunit (gray) at the ECD interface, highlighted in zoom-in views **a** and **b**. **c** Interaction network of the triad D32-R192-D122 at the intersubunit interface, located on the β1-β2 loop, pre-M1 loop, and Pro-loop, respectively. Interacting residues are shown as sticks. Interactions are shown as dashed lines and the corresponding distances are labeled.

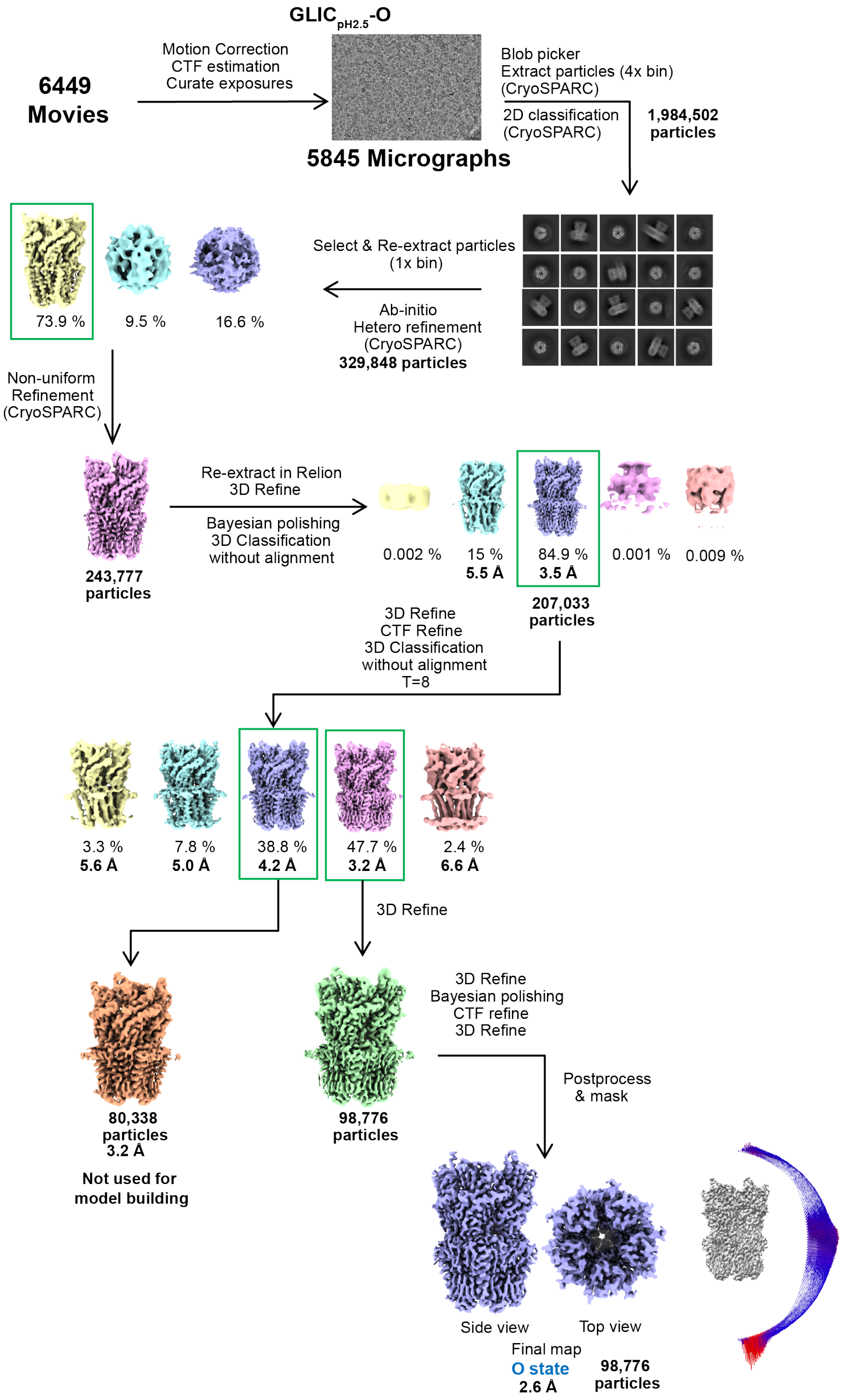

Supplementary figure 12. Cryo-EM data processing workflow of GLIC at pH 2.5.

Cryo-EM data processing workflow of $GLIC_{pH2.5}$-O showing representative micrograph and 2D classes and angular distribution. Systematic data processing, including iterative 2D, 3D classification and refinement, led to state O with nominal resolution of 2.6 Å.

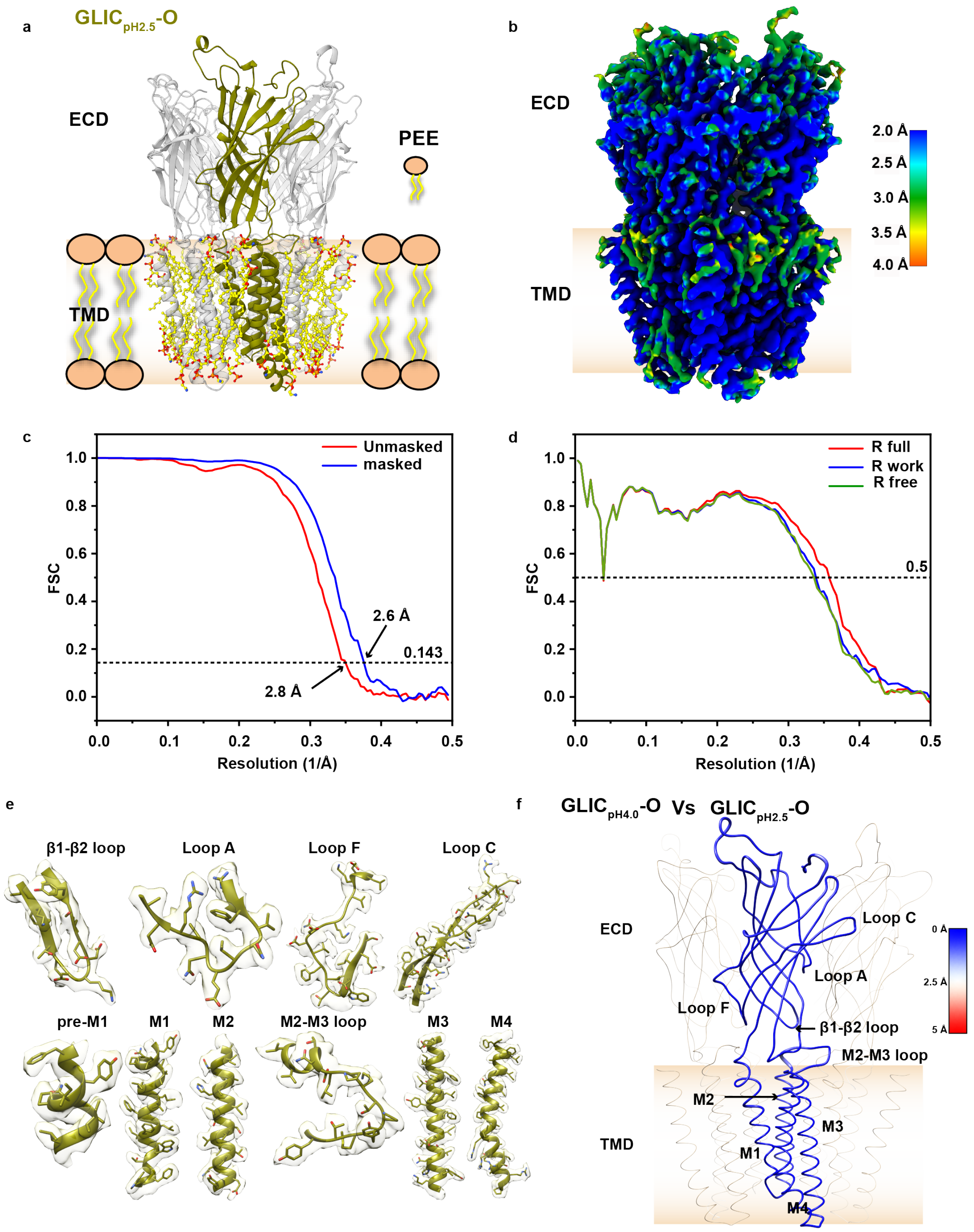

Supplementary figure 13. Cryo-EM data quality assessment and model validation of GLIC reconstituted in nanodiscs at pH 2.5 in state O. a Overall structure of $GLIC_{pH2.5}$-O. One subunit is colored olive and the other subunits are colored gray. Lipids (PEE:

phosphatidylethanolamine, yellow) are shown as balls and sticks and as schematics in the membrane bilayer. **b** Side view of 3D reconstructed map of the corresponding structure is colored by the local resolution calculated using the ResMap program. A color key corresponding to the resolution is also inserted. A schematic of bilayer is shown as a gradient of apricot color. **c** The gold standard Fourier shell correlation (FSC) curves before (red) and after (blue) using the mask are shown for the respective structures. The dashed line represents an FSC of 0.143. **d** Model Vs map validation FSC curves for the corresponding structure are shown. For cross validation of model calculation, the FSC curves of the refined model versus the summed map (R_{full}, red), refined model versus half map 1 (used during refinement, R_{work}, blue), and refined model versus half map 2 (not used during refinement, R_{free}, green) are plotted. **e** Validation of various regions is shown by extracting density maps of critical regions (transparent volume in yellow). The corresponding region of the model is depicted as a cartoon with sticks. **f** Pairwise comparisons of $GLIC_{pH4.0}$-O with $GLIC_{pH2.5}$-O show minimal conformational changes. The principal subunit is represented as putty and is colored by RMSD. Other subunits are represented in licorice (tan). The RMSD color code and ribbon thickness scale are inserted. The membrane bilayer is shown as a gradient of apricot color.

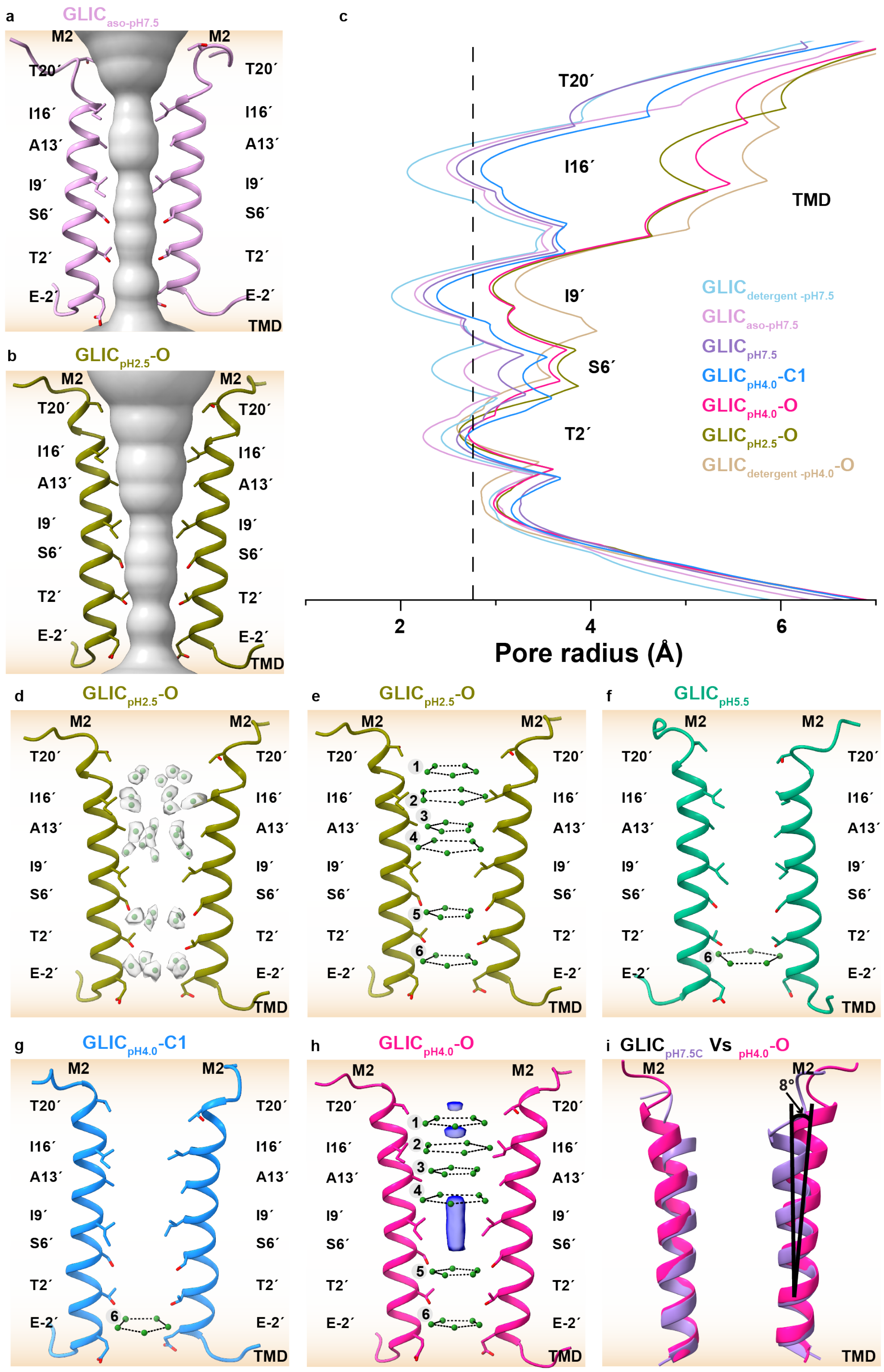

Supplementary figure 14. The ion permeation pathway. Pore profiles (surface representation in gray) were calculated using the HOLE program for **a** GLIC$_{aso\text{-}pH7.5}$ (plum) and **b** GLIC$_{pH2.5}$-O (olive). The M2 helices from the two diagonal subunits are shown in

cartoon for clarity, and the pore-lining residues are shown as sticks. **c** Comparison of the pore radius plotted against the distance along the pore axis. The color key for pore radius is as indicated in the plot. The vertical black dotted line represents the approximate radius of the hydrated Na^+ ion. **d** The cryo-EM density (gray, transparent) of water molecules (green) observed in $GLIC_{pH2.5}$-O is shown. **e** Six pentagons of water molecules (green) observed in the pore of $GLIC_{pH2.5}$-O. **f, g** A water pentagon observed in $GLIC_{pH5.5}$ (green) and $GLIC_{pH4.0}$-C1 (dodger blue) is denoted as spheres (green) connected by dashed lines. **h** The cryo-EM density observed at the pore axis in $GLIC_{pH4.0}$-O (deep pink) is shown as surface (blue, transparent). The water molecules are shown as spheres (green) connected by dashed lines. **i** Superposition of M2 helix of $GLIC_{pH7.5}$ (purple) and $GLIC_{pH4.0}$-O (deep pink) shows rotation of 8° in M2 helix. The M2 helices from the two subunits are shown for clarity. The membrane bilayer is shown as a gradient of apricot color.

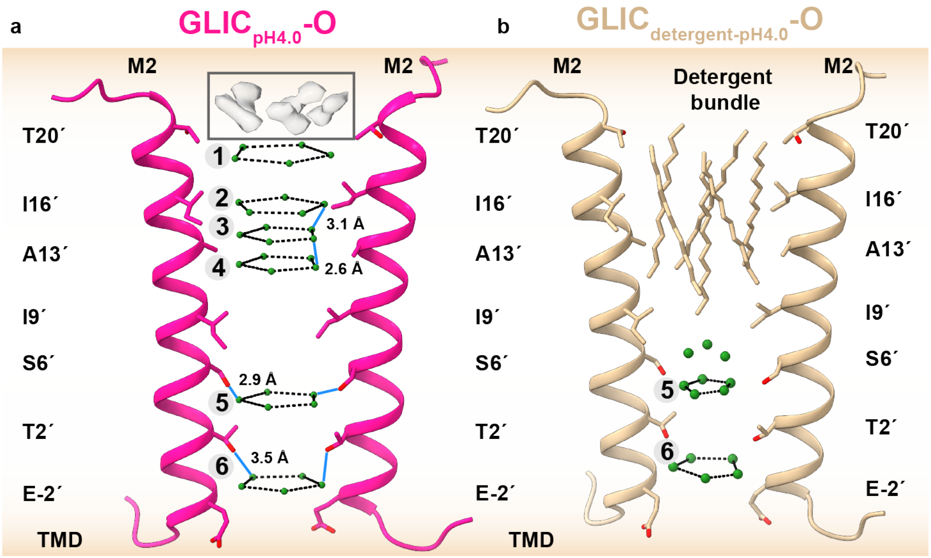

Supplementary figure 15. Water pentagons and detergent bundle in GLIC open state. **a** Six pentagons (1-6) of water molecules (green) observed in the pore of GLIC$_{pH4.0}$-O. Water molecules in pentagons 2-4 are stabilized by interaction among layers. Additional non-protein densities above the pentagon 1 are shown as gray volumes inside the box. No clear interactions are observed involving water molecules in pentagon 1. Water molecules in pentagons 5-6 are stabilized by direct interaction with polar residues located at the M2 helices. **b** The detergent bundle (sticks) and water pentagons (5-6) observed in the pore of GLIC$_{detergent\text{-}pH4.0O}$ are shown. The M2 helices from two diagonal subunits are shown for clarity, and the pore-lining residues are shown as sticks.

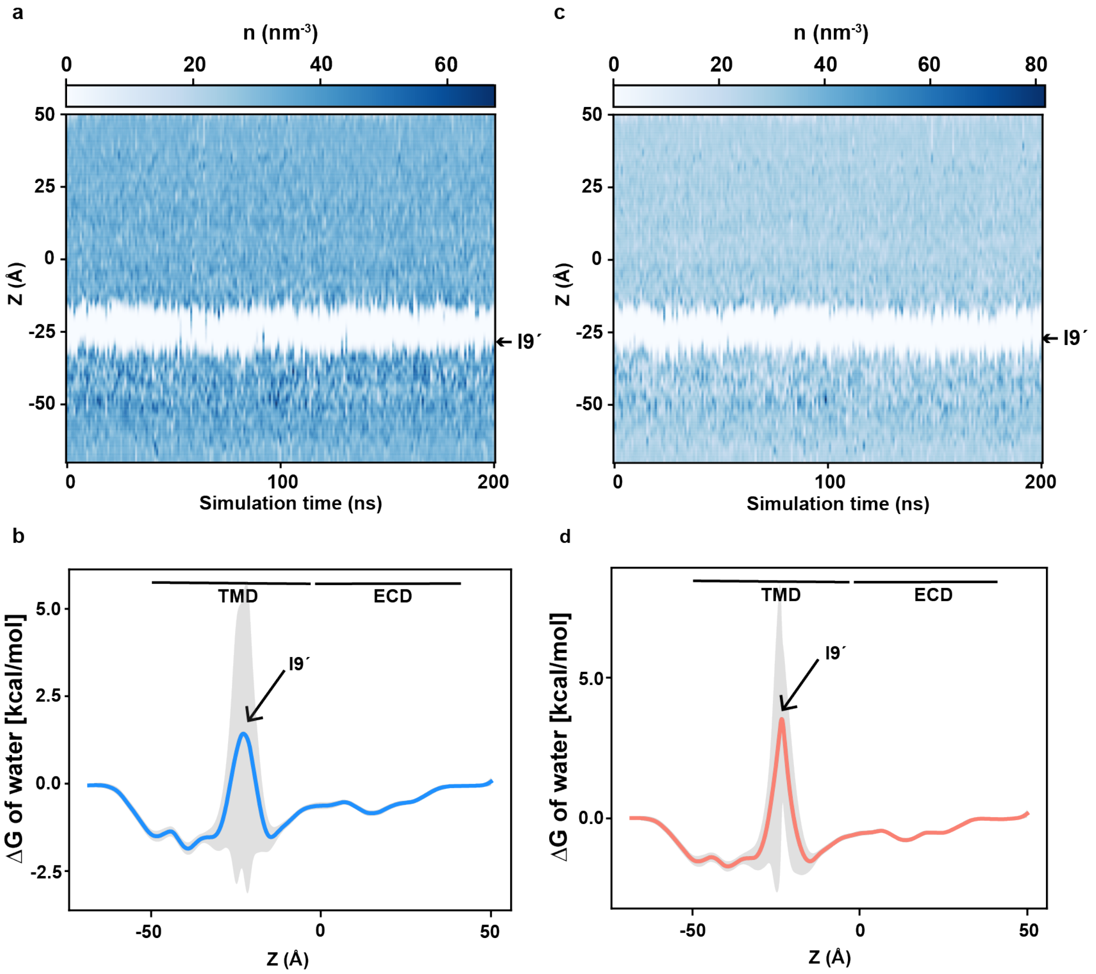

Supplementary figure 16. Molecular dynamics simulations to investigate the pore hydration profile for C1 and C2 states at pH 4.0. a, c Time series of water molecules are plotted for a simulation time of 200 ns for each corresponding structures. White stretches reflect regions lacking water. **b, d** Free-energy profiles of a water molecule along the central axis are shown for the C1 (dodger blue) and C2 (salmon) states.

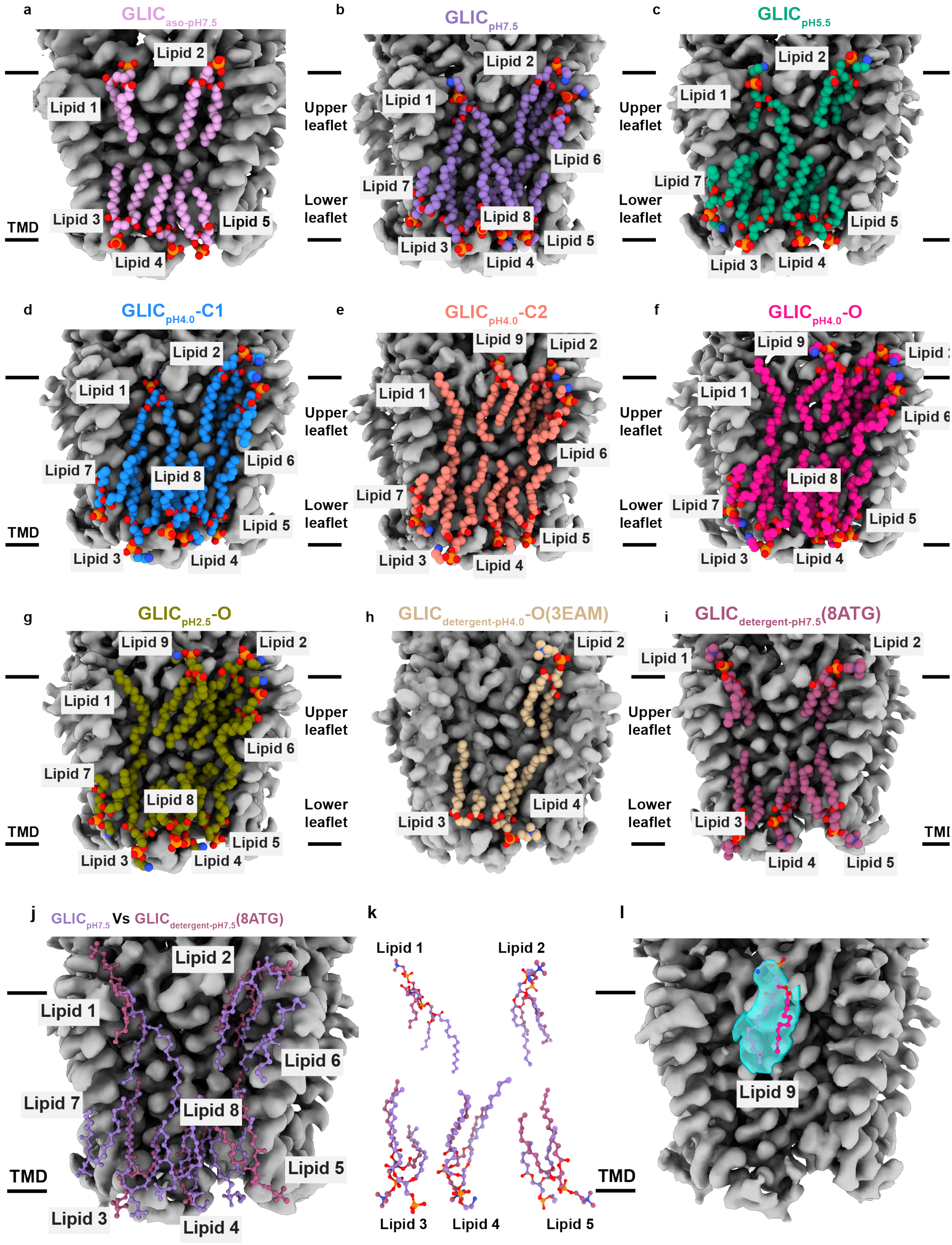

Supplementary figure 17. Lipids bound to GLIC at various pH. Lipids bound to **a** GLIC$_{aso\text{-}pH7.5}$ (plum), **b** GLIC$_{pH7.5}$ (purple), **c** GLIC$_{pH5.5}$ (green), **d** GLIC$_{pH4.0}$-C1 (dodger blue), **e** GLIC$_{pH4.0}$-C2 (salmon), **f** GLIC$_{pH4.0}$-O (deep pink), **g** GLIC$_{pH2.5}$-O (olive), **h**

$GLIC_{detergent\text{-}pH4.0O}$ (3EAM) (tan) and **i** $GLIC_{detergent\text{-}pH7.5}$ (8ATG) (royal heath) are shown as spheres. **j** Comparison of all lipid (ball-stick) binding locations between $GLIC_{pH7.5}$ and $GLIC_{detergent\text{-}pH7.5}$ (8ATG). Cryo-EM maps are shown in gray. The map for 3EAM is generated from the molecular model as a representative of open structures solved by X-ray crystallography. **k** Comparison of Lipids 1-5 positions between $GLIC_{pH7.5}$ and $GLIC_{detergent\text{-}pH7.5}$ (8ATG). **l** Lipid 9 of $GLIC_{pH4.0}$-O is superimposed on the predicted occupancy derived map at a similar location in $GLIC_{detergent\text{-}pH7.5}$ (8ATG).

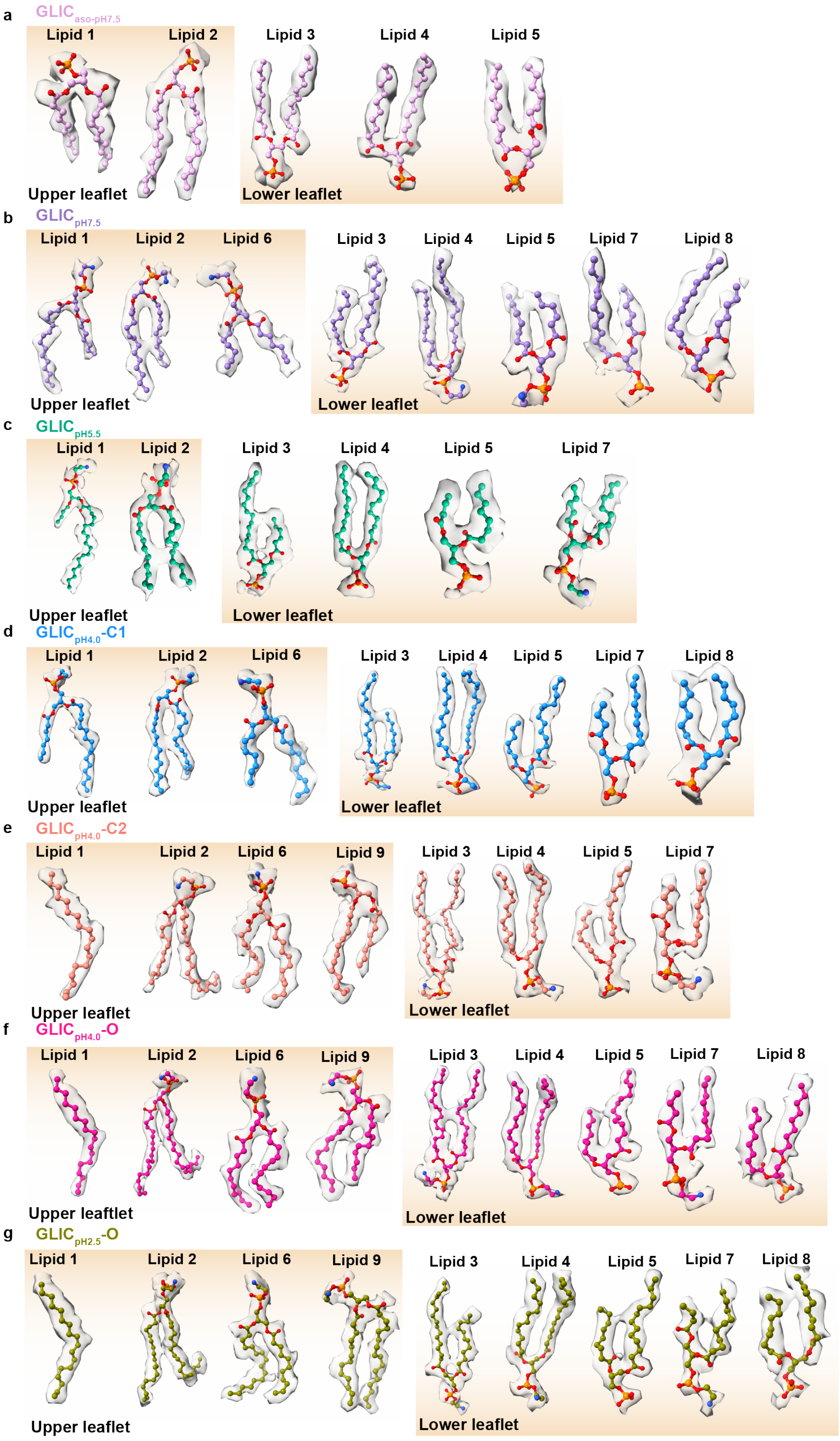

Supplementary figure 18. Validation of lipids built into cryo-EM maps. Cryo-EM density maps for lipids bound to **a** GLIC$_{aso\text{-}pH7.5}$ (plum), **b** GLIC$_{pH7.5}$ (purple), **c** GLIC$_{pH5.5}$ (green), **d** GLIC$_{pH4.0}$-C1 (dodger blue), **e** GLIC$_{pH4.0}$-C2 (salmon), **f** GLIC$_{pH4.0}$-O (deep pink),

g $GLIC_{pH2.5}$-O (olive) are extracted and shown as a surface (transparent, gray). Lipids are shown as balls and sticks, and they are segregated according to their location in upper or lower leaflet shown as a gradient of apricot color.

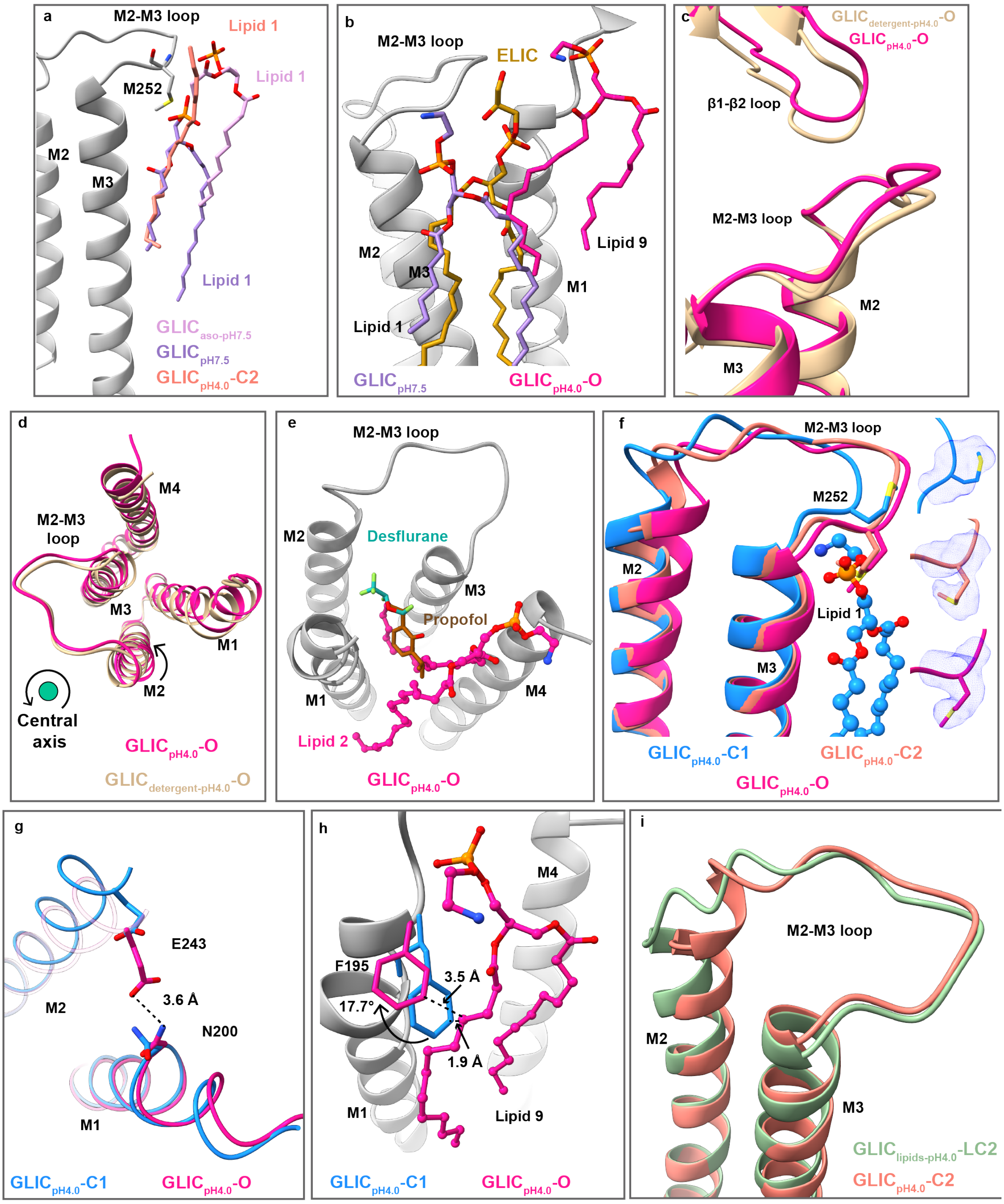

Supplementary figure 19. The state dependent positional fluctuations of lipids in GLIC. **a** Comparison of Lipid 1 bound to $GLIC_{aso\text{-}pH7.5}$ (plum), $GLIC_{pH7.5}$ (purple), and $GLIC_{pH4.0}$-C2 (salmon), respectively. M252 and lipids are shown as sticks. **b** A lipid in the ELIC

structure (yellow ochre) occupies a position between the locations of Lipid 1b and Lipid 9 of $GLIC_{pH7.5}$ (purple) and $GLIC_{pH4.0}$-O (deep pink), respectively. **c** Superposition of β1-β2 loop and M2–M3 loop of $GLIC_{detergent\text{-}pH4.0O}$ (tan) and $GLIC_{pH4.0}$-O (deep pink) showing conformational changes. **d** A subtle counter-clockwise rotation of the upper part of M2 is observed in $GLIC_{detergent\text{-}pH4.0O}$ (tan) compared with $GLIC_{pH4.0}$-O (deep pink). TMD of only one subunit is shown in the top view for clarity. **e** Lipid 2 of $GLIC_{pH4.0}$-O (deep pink) wedges in the cavity formed by the M1, M3 and M4 helices (gray). Desflurane and propofol occupy the same cavity. Lipid 2 is shown as balls and sticks, whereas desflurane (sea green) and propofol (brown) are shown as sticks. **f** The position of M252 in $GLIC_{pH4.0}$-C2 (salmon) and $GLIC_{pH4.0}$-O (deep pink) sterically clashed with Lipid 1 of $GLIC_{pH4.0}$-C1 (dodger blue). Cryo-EM density of M252 in all three structures is shown. Lipid is depicted as balls and sticks, whereas M252 as sticks. M2 and M3 are shown as cartoons. **g** E243 located at the upper part of M2 in the principal (+) subunit interacts with N200 of the complementary (+) subunit in $GLIC_{pH4.0}$-O (deep pink) but not in $GLIC_{pH4.0}$-C1 (dodger blue). Interacting residues are shown as sticks, and the M1 and M2 helices are shown in licorice representation. **h** The rotation of F195 (17.7°) from C1 state (dodger blue) leads to the binding of Lipid 9 (deep pink) at C2 and O (both pH4.0 and 2.5) states. Lipid 9 (deep pink) present in the O state of $GLIC_{pH4.0}$ is shown as a representative. Lipid 9 is shown as balls and sticks, and F195 is shown as sticks. The protein is represented as a cartoon. Distance between F195 and Lipid 9 is labeled. **i** Comparison of M2–M3 loop of $GLIC_{detergent\text{-}pH4.0LC2}$ (sea green, PDB ID: 3TLS) and $GLIC_{pH4.0}$-C2 (salmon).

Supplementary Table 1: Data collection/processing

	GLIC$_{aso-pH7.5}$ EMDB-35161 PDB-8I41	GLIC$_{pH7.5}$ EMDB-35162 PDB 8I42	GLIC$_{pH5.5}$ EMDB-35163 PDB-8I47	GLIC$_{pH4.0}$-C1 EMDB-35164 PDB-8I48	GLIC$_{pH4.0}$-C2 EMDB-37446 PDB-8WCQ	GLIC$_{pH4.0}$-O EMDB-37447 PDB-8WCR	GLIC$_{pH2.5}$-O EMDB-36339 PDB-8JJ3
**Data Collection and processing**							
Magnification	130,000x	165,000x	165,000x		165,000x		165,000x
Voltage (kV)	300	300	300		300		300
Image filter	BioQuantum	SelectrisX	BioQuantum		SelectrisX		BioQuantum
Slit width (eV)	20	10	20		10		20
Electron exposure ($e^-/Å^2$)	40	71	57		71		74
Defocus range (µm)	-1.0 to -2.0	-0.6 to -1.4	-0.8 to -1.6		-0.6 to -1.4		-0.6 to -2.0
Pixel size (Å)	1.064	0.76	0.8452		0.76		0.8584
Symmtry imposed	C5	C5	C5		C5		C5
Micrographs	5,188	14,001	6,625		14,076		6,449
Initial particle images (no.)	635,408	1,577,434	365,137		1,071,249		325,848
Final particle images (no.)	59,086	152,938	61,526	254,591	78,444	178,543	98,776
Map resolution at 0.143 FSC (Å)	3.4	2.9	2.7	2.7	3.3	2.7	2.6
Map sharpening *B* factor ($Å^2$)	-35	-30	-60	-30	-40	-30	-30
**Refinement**							
Initial model used (PDB code)	4NPQ	8I41	8I41	8I41	8I41	8I41	8I41
Model composition							
Non-hydrogen atoms	13,515	14,060	13,695	14,270	14,130	14,615	14,635
Protein residues	1,565	1,560	1,560	1,560	1,560	1,560	1,560
Ligands	25	40	30	40	40	45	45
CC mask	0.85	0.84	0.85	0.85	0.86	0.9	0.89
B factor ($Å^2$)							
Protein residues	109.7	134.66	106.38	106.99	71.75	57.64	61.65
Ligands	106.61	122.36	87.72	109.24	73.3	98.67	89.35
R.M.S. deviations							
Bond lengths (Å)	0.002	0.002	0.004	0.005	0.003	0.005	0.005

Bond angles (°)	0.474	0.532	0.841	0.885	0.522	0.813	0.882
Validation							
MolProbity score	1.71	1.95	1.93	1.94	1.44	1.41	1.78
Clashscore	5.38	5.50	11.54	12.90	5.98	4.48	5.09
Poor rotamers (%)	0.36	2.14	1.07	0.36	0	1.07	2.14
Ramachandran plot							
Favored (%)	93.57	94.52	95.16	95.48	97.42	97.10	96.13
Allowed (%)	6.43	5.48	4.84	4.52	2.58	2.90	3.87
Disallowed (%)	0	0	0	0	0	0	0
