# Supplementary material for: Cryo-EM structures of prokaryotic ligand-gated ion channel GLIC provide insights into gating in a lipid environment
Source: Nat Commun. 2024 Apr 5;15:2967. doi: 10.1038/s41467-024-47370-w (PMC10997623; doi:10.1038/s41467-024-47370-w)
Supplement: Supplementary file 3 — Description of Additional Supplementary Files [file 41467_2024_47370_MOESM3_ESM.pdf]

## Description of Additional Supplementary Files

**Supplementary Movie 1. GLIC undergoing overall conformational changes from the resting-like state (purple) to the open state (deep pink).** The ECD and TMD of GLIC are labeled. In the ECD, a compaction arises first, which is sequentially followed by the counter-clockwise rotation toward open state.

**Supplementary Movie 2. Conformational changes in TMD of GLIC from the resting-like state (purple) to the open state (pink).** The extracellular side of TMD is displayed, with the M2 helices labeled. During activation, M2-M3 loop moves outward followed by counter-clockwise rotation of M2 helices resulting in channel opening.

**Supplementary Movie 3. The cascade of conformational changes from the resting-like state to the open state highlighting important regions.** The overall structure of $GLIC_{pH7.5}$ is depicted. Only two diagonal subunits are shown for clarity. The conformational changes in the Loop A, β1- β2 loop, and Loop F are shown in succession, with the pH 5.5 model, C1, C2 and O superimposed onto the pH 7.5 model. Finally, a top view of the TMD highlights the conformational change of M2–M3 loop and M2 helix in all five structures.
