# Supplementary material for: Cryo-EM structures of prokaryotic ligand-gated ion channel GLIC provide insights into gating in a lipid environment
Source: Nat Commun. 2024 Apr 5;15:2967. doi: 10.1038/s41467-024-47370-w (PMC10997623; doi:10.1038/s41467-024-47370-w)
Supplement: Supplementary file 7 — Reporting Summary [file 41467_2024_47370_MOESM7_ESM.pdf]

nature portfolio

Corresponding author(s): Sandip Basak

Last updated by author(s): Mar 8, 2024

# Reporting Summary

Nature Portfolio wishes to improve the reproducibility of the work that we publish. This form provides structure for consistency and transparency in reporting. For further information on Nature Portfolio policies, see our Editorial Policies and the Editorial Policy Checklist.

## Statistics

For all statistical analyses, confirm that the following items are present in the figure legend, table legend, main text, or Methods section.

| n/a | Confirmed | |
|---|---|---|
| ☐ | ☒ | The exact sample size (*n*) for each experimental group/condition, given as a discrete number and unit of measurement |
| ☐ | ☒ | A statement on whether measurements were taken from distinct samples or whether the same sample was measured repeatedly |
| ☒ | ☐ | The statistical test(s) used AND whether they are one- or two-sided<br>*Only common tests should be described solely by name; describe more complex techniques in the Methods section.* |
| ☒ | ☐ | A description of all covariates tested |
| ☒ | ☐ | A description of any assumptions or corrections, such as tests of normality and adjustment for multiple comparisons |
| ☐ | ☒ | A full description of the statistical parameters including central tendency (e.g. means) or other basic estimates (e.g. regression coefficient) AND variation (e.g. standard deviation) or associated estimates of uncertainty (e.g. confidence intervals) |
| ☒ | ☐ | For null hypothesis testing, the test statistic (e.g. *F*, *t*, *r*) with confidence intervals, effect sizes, degrees of freedom and *P* value noted<br>*Give P values as exact values whenever suitable.* |
| ☒ | ☐ | For Bayesian analysis, information on the choice of priors and Markov chain Monte Carlo settings |
| ☒ | ☐ | For hierarchical and complex designs, identification of the appropriate level for tests and full reporting of outcomes |
| ☒ | ☐ | Estimates of effect sizes (e.g. Cohen's *d*, Pearson's *r*), indicating how they were calculated |

*Our web collection on statistics for biologists contains articles on many of the points above.*

## Software and code

Policy information about availability of computer code

| | |
|---|---|
| Data collection | EPU v3.3.1.5184REL from ThermoFisher; SerialEM v4.0.6 |
| Data analysis | 1. Drift correction: MotionCor2 version 1.6.3<br>2. CTF estimation: Ctffind version 4.1.13<br>3. Particle picking: TOPAZ v0.2.5a<br>4. 2D and 3D Reconstruction, 3D refinement, post-processing: Relion Version 4.0.1, CryoSPARC Version4.3.1<br>5. Local resolution estimation: Resmap version 1.1.4<br>6. Pore profile calculation: HOLE version v2.2.005<br>7. Model visualization: ChimeraX v1.6.1<br>8. 3D volume visualization: Chimera version 1.17, Chimerax 1.6.1<br>9. Manual model building: Coot 0.9.8.7<br>10. Structure refinement: Phenix version 1.21rc1-4985<br>11. MD simulations: CHARMM-GUI v3.8, GROMACS 2021, CHARMM36m, Channel Annotation Package v0.9.1,<br>12. Various CRYO-EM data conversion: EMAN version 2.99.47<br>13. Particle export from cryoSPARC to Relion: pyem v2.1.0<br>14. Figure generation: Adobe Illustrator 27.9<br>15. FSC plot generation: OriginPro v9.9.0.225<br>16. Protein surface area and interfaces calculations: CCP4i2 (v1.0.2)<br>17. Stereochemistry analysis: MolProbity web server (v4.5.2) |

For manuscripts utilizing custom algorithms or software that are central to the research but not yet described in published literature, software must be made available to editors and reviewers. We strongly encourage code deposition in a community repository (e.g. GitHub). See the Nature Portfolio guidelines for submitting code & software for further information.

## Data

Policy information about availability of data

All manuscripts must include a data availability statement. This statement should provide the following information, where applicable:

- Accession codes, unique identifiers, or web links for publicly available datasets
- A description of any restrictions on data availability
- For clinical datasets or third party data, please ensure that the statement adheres to our policy

The coordinates of the PDBs and EMDB maps are provided.
Atomic structures are deposited with PDB IDs:

| | | |
|---|---|---|
| GLIC-aso-pH7.5C | 8I41 | https://www.rcsb.org/structure/8I41 |
| GLIC-lipids-pH7.5C | 8I42 | https://www.rcsb.org/structure/8I42 |
| GLIC-lipids-pH5.5C | 8I47 | https://www.rcsb.org/structure/8I47 |
| GLIC-lipids-pH4.0C1 | 8I48 | https://www.rcsb.org/structure/8I48 |
| GLIC-lipids-pH4.0C2 | 8WCQ | https://www.rcsb.org/structure/8WCQ |
| GLIC-lipids-pH4.0O | 8WCR | https://www.rcsb.org/structure/8WCR |
| GLIC-lipids-pH2.5O | 8JJ3 | https://www.rcsb.org/structure/8JJ3 |

Corresponding maps are deposited with EMDB IDs:

| | |
|---|---|
| EMD-35161 | https://www.ebi.ac.uk/emdb/EMD-35161 |
| EMD-35162 | https://www.ebi.ac.uk/emdb/EMD-35162 |
| EMD-35163 | https://www.ebi.ac.uk/emdb/EMD-35163 |
| EMD-35164 | https://www.ebi.ac.uk/emdb/EMD-35164 |
| EMD-37446 | https://www.ebi.ac.uk/emdb/EMD-37446 |
| EMD-37447 | https://www.ebi.ac.uk/emdb/EMD-37447 |
| EMD-36339 | https://www.ebi.ac.uk/emdb/EMD-36339 |

The MD data are uploaded to zenodo: https://doi.org/10.5281/zenodo.10792701

## Research involving human participants, their data, or biological material

Policy information about studies with human participants or human data. See also policy information about sex, gender (identity/presentation), and sexual orientation and race, ethnicity and racism.

| | |
|---|---|
| Reporting on sex and gender | N/A |
| Reporting on race, ethnicity, or other socially relevant groupings | N/A |
| Population characteristics | N/A |
| Recruitment | N/A |
| Ethics oversight | N/A |

Note that full information on the approval of the study protocol must also be provided in the manuscript.

# Field-specific reporting

Please select the one below that is the best fit for your research. If you are not sure, read the appropriate sections before making your selection.

☒ Life sciences ☐ Behavioural & social sciences ☐ Ecological, evolutionary & environmental sciences

For a reference copy of the document with all sections, see nature.com/documents/nr-reporting-summary-flat.pdf

# Life sciences study design

All studies must disclose on these points even when the disclosure is negative.

| | |
|---|---|
| Sample size | No explicit sample size calculations were implemented for the cryo-EM studies in this work. The number of micrographs and particles are sufficient for obtaining reliable classifications and reconstructions as indicated by the data processing. |
| Data exclusions | Particles with lesser quality were excluded by iterative 2D and 3D classifications as is generally endorsed by cryo-EM studies. |
| Replication | No replications were applied in this work. In cryo-EM studies, statistical analyses and validation procedures are generally sufficient to evaluate the quality and reproducibility of cryo-EM results. |

| | |
|---|---|
| Randomization | As is generally demanded by cryo-EM studies, randomization was performed at 3D reconstruction stage for the calculation of FSC curves illustrated in supplementary figures. |
| Blinding | Blinding is not generally feasible for structural studies as a specific protein subject is being studied in a defined environment/condition. |

# Reporting for specific materials, systems and methods

We require information from authors about some types of materials, experimental systems and methods used in many studies. Here, indicate whether each material, system or method listed is relevant to your study. If you are not sure if a list item applies to your research, read the appropriate section before selecting a response.

## Materials & experimental systems

| n/a | Involved in the study |
|---|---|
| ☒ | ☐ Antibodies |
| ☒ | ☐ Eukaryotic cell lines |
| ☒ | ☐ Palaeontology and archaeology |
| ☒ | ☐ Animals and other organisms |
| ☒ | ☐ Clinical data |
| ☒ | ☐ Dual use research of concern |
| ☒ | ☐ Plants |

## Methods

| n/a | Involved in the study |
|---|---|
| ☒ | ☐ ChIP-seq |
| ☒ | ☐ Flow cytometry |
| ☒ | ☐ MRI-based neuroimaging |

# Plants

| | |
|---|---|
| Seed stocks | N/A |
| Novel plant genotypes | N/A |
| Authentication | N/A |
